# Modelling Vector Transmission and Epidemiology of Co-Infecting Plant Viruses

**DOI:** 10.3390/v11121153

**Published:** 2019-12-13

**Authors:** Linda J. S. Allen, Vrushali A. Bokil, Nik J. Cunniffe, Frédéric M. Hamelin, Frank M. Hilker, Michael J. Jeger

**Affiliations:** 1 Department of Mathematics and Statistics, Texas Tech University, Lubbock, TX 79409, USA; 2 Department of Mathematics, Oregon State University, Corvallis, OR 97331, USA; bokilv@math.oregonstate.edu; 3 Department of Plant Sciences, University of Cambridge, Cambridge CB2 3EA, UK; njc1001@cam.ac.uk; 4 IGEPP, Agrocampus Ouest, INRA, Université de Rennes 1, Université Bretagne-Loire, 35000 Rennes, France; frederic.hamelin@agrocampus-ouest.fr; 5 Institute of Environmental Systems Research, School of Mathematics/Computer Science, Osnabrück University, 49069 Osnabrück, Germany; frank.hilker@uni-osnabrueck.de; 6 Centre for Environmental Policy, Imperial College London, Ascot SL5 7PY, UK; m.jeger@imperial.ac.uk

**Keywords:** co-infection, invasion reproduction number, vector transmission

## Abstract

Co-infection of plant hosts by two or more viruses is common in agricultural crops and natural plant communities. A variety of models have been used to investigate the dynamics of co-infection which track only the disease status of infected and co-infected plants, and which do not explicitly track the density of inoculative vectors. Much less attention has been paid to the role of vector transmission in co-infection, that is, acquisition and inoculation and their synergistic and antagonistic interactions. In this investigation, a general epidemiological model is formulated for one vector species and one plant species with potential co-infection in the host plant by two viruses. The basic reproduction number provides conditions for successful invasion of a single virus. We derive a new invasion threshold which provides conditions for successful invasion of a second virus. These two thresholds highlight some key epidemiological parameters important in vector transmission. To illustrate the flexibility of our model, we examine numerically two special cases of viral invasion. In the first case, one virus species depends on an autonomous virus for its successful transmission and in the second case, both viruses are unable to invade alone but can co-infect the host plant when prevalence is high.

## 1. Introduction

Transmission is a key element in understanding the epidemiology of plant virus diseases, particularly those transmitted by arthropod vectors [1,2,3,4]. In general, four modes of transmission, non-persistent, semi-persistent, persistent-circulative and persistent-propagative, can be distinguished. Each of these modes has a characteristic time period for acquisition from infected plants, retention in the vector, and inoculation to healthy plants [5], although some virus groups such as the torradoviruses do not fit neatly into these categories [6]. Other aspects important for arthropod transmission include transovarial and transtadial transmission, and the “helper strategy” [3] in which a helper virus can be transmitted by the vector but the dependent virus can only be transmitted in the presence of the helper, a strategy modelled by Zhang et al. [7].

Co-infection of hosts by two or more plant viruses is common in both agricultural crops [8,9] and natural plant communities [10,11]. Because of this, the literature on co-infection by plant viruses is extensive, although often not related to transmission. Indeed, extending epidemiological models to go beyond a single pathogen species was relatively-recently highlighted as a key challenge in modeling plant diseases in [12] (challenge 4). Co-infection almost always leads to interactions between viruses during transmission and within-plant processes that can strongly influence disease development in individual plants and ultimately spread in a plant population. The strength and direction of interactions can vary with both negative and facilitating effects, involving within-cell processes, cell-to-cell movement, vector acquisition and inoculation, symptom development and virulence, and yield loss. Reports on cellular interactions have been the most prevalent, mostly for replication rates and virus titre, but some studies have shown clear interactions with vectors over short (epidemiological) and long-term (evolutionary) time scales [13].

Most experimental studies on the relationship between co-infection and transmission have been done for viruses with non-persistent transmission by aphids. Syller [14] reviewed the literature on “simultaneous” transmission of plant viruses by vectors, emphasizing the acquisition component of transmission with little consideration of inoculation. The use of the term “simultaneous” is ambiguous—what seems to be suggested is that two different virus particles can be acquired instantaneously by a vector during a single probe where there is spatial separation between two viruses. However, for non-persistent transmission, with a following probe [15] on the same or different plant, one of the viruses can become detached and no longer be available for inoculation. Transmission can present a real bottleneck in the virus life cycle [16]. In a subsequent review, Syller and Grupa [17] differentiate between simultaneous inoculation (which they call co-infection) and sequential inoculation (which they call super-infection). They claim that synergistic interactions within-plants most often arise between unrelated viruses. Synergism is defined as a facilitative effect in which accumulation of one or both viruses in the host plant increases; in the case of the effect on just one virus, it has been called asymmetric synergism [18]. Synergism has also been used to describe more severe disease symptoms than induced by either virus alone. Syller and Grupa [17] concentrate more on antagonistic effects, such as cross protection [19] or, as has been termed “super-infection exclusion” in which related viruses or virus strains are used preventively to exclude more virulent strains. Mascia and Gallitelli [20] note the contributions that mathematical modeling could make “in forecasting challenges deriving from the great variety of pathways of synergistic and antagonistic interactions” (p. 176).

Co-infection can cover scenarios ranging from two viruses (or virus strains)/one vector through to many viruses/many vectors, but with some nuances. There is an extensive literature on co-infection across this range. Some representative but not exhaustive publications are noted in Table 1, together with some key messages. Many publications acknowledge that there are several or many (in the case of aphids) vector species for a given plant virus, but the experiments reported only involve one vector. Similarly, the same virus and vector can infect more than one host (cucumber mosaic virus is an extreme example) and hence cause more than one disease. Co-infection with virus strains differing in virulence (or other characteristic) can lead to the same set of interactions and consequences as found with virus species. A good example of two strains of the same virus species with shared vector species is potato virus Y (PVY) [21,22,23] on potato and other hosts [15]. There are many examples of two co-infecting virus species with a shared vector species [24,25,26]. Similarly, there are many cases where two co-infecting viruses have quite different vectors taxonomically [27]. Co-infection is manifested in more complex situations with multiple viruses and vectors such as with grapevine leafroll disease [28,29] and sweet potato virus disease (SPVD) [30]. At an even higher level of complexity, the ecological networks formed by multiple co-infecting viruses and multiple hosts were analyzed by McLeish et al. [31].

In this paper, we formulate a general epidemiological model for one vector species and one plant species that allows for co-infection of the host plant by two virus species or strains. The model is used to investigate the role of vector transmission on co-infection, specifically acquisition and inoculation, as well as antagonistic and synergistic interactions. The basic reproduction number provides a condition for invasion of a single virus infection. For co-infection, we derive a new invasion threshold. Given that a single virus can persist in the host plant, the invasion threshold highlights some key epidemiological parameters for successful co-infection. In addition, we investigate the roles of the vector acquisition and inoculation parameters when one virus depends on an autonomous virus for its successful transmission, or when both viruses are unable to invade alone but can facilitate co-infection if they occur in high enough prevalences. We also explicitly test when the simplifications—which are almost always left implicit—in models which do not explicitly include the infection status of vectors lead to potentially misleading results.

## 2. Materials and Methods

### 2.1. Modelling

**Figure 1:**
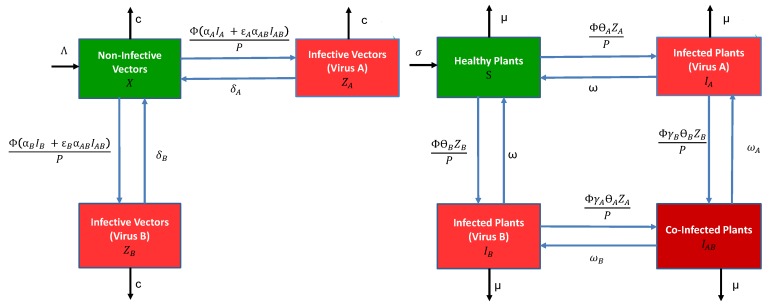
Compartmental diagrams (**left**) for the vector model and (**right**) the host plant model, described by the differential Equations (1) and (2), respectively. Meanings of the symbols for model parameters are summarised in Table 2.

The general epidemiological model for vectors and plants consists of a system of differential equations with either infection by a single virus, or co-infection by two viruses (species or strains). For simplicity, we refer to the two viruses as virus *A* and virus *B*. We make several simplifying assumptions. The two viruses are not transmitted vertically in the plant population (no transmission by seeds or other propagating material) nor in the vector (no transovarial transmission). The plants can be infected by a single virus or co-infected by both viruses. The vectors are only inoculative with a single virus; namely, we assume that acquisition of the first virus precludes inoculation by a second virus by the same vector for as long as the vector retains the first virus. This assumption applies to both modes of transmission. For non-persistent transmission, it may arise because stylet receptor sites are saturated by the first virus. For persistent-circulative transmission, it may be due to a phenomenon similar to “super-infection exclusion” taking place in the vector [16], or simply that the first virus moves back to the salivary glands faster than the second. In addition, the latent stages in the vector and plant are ignored. The following compartmental diagrams in Figure 1 illustrate the rates of change between the vector and plant stages. The vector and plant models are described in more detail below.

The vector model has three stages, X= the density of non-infective vectors, $Z_{A}=$ the density of infective vectors carrying virus *A* and $Z_{B}=$ the density of infective vectors carrying virus *B* (for vectors, “infective” more accurately means “inoculative”). The total vector density is $V=X+Z_{A}+Z_{B}$. The parameters Λ= net vector birth rate, c= per capita vector death rate, Φ= number of plants visited per unit time by a single vector, $\alpha_{i}=$ probability a non-infective vector acquires a single virus from an infected plant $I_{i}$, i=A,B,AB, per plant visit and $\delta_{i}=$ per capita vector recovery rate from virus i=A,B. The parameters $\epsilon_{A}$ and $\epsilon_{B}$ multiplying the acquisition probability $\alpha_{AB}$ are the conditional probabilities that either virus *A* or *B* are acquired, given that acquisition is from a co-infected plant, $\epsilon_{A}+\epsilon_{B}=1$. All of the parameters are non-negative. They are summarised in Table 2. With these assumptions, as well as the assumption of frequency-dependent transmission with *P* being the total plant population density, the vector model takes the following form:

(1) $$\mathrm{Vector}\;\left\{\begin{array}{cc} \frac{dX}{dt} & =\Lambda -\Phi X\left[\alpha_{A}I_{A}+\alpha_{B}I_{B}+\alpha_{AB}I_{AB}\right]/P-cX+\delta_{A}Z_{A}+\delta_{B}Z_{B}\;\\ \frac{dZ_{A}}{dt} & =\Phi X\left[\alpha_{A}I_{A}+\epsilon_{A}\alpha_{AB}I_{AB}\right]/P-\left(c+\delta_{A}\right)Z_{A}\;\\ \frac{dZ_{B}}{dt} & =\Phi X\left[\alpha_{B}I_{B}+\epsilon_{B}\alpha_{AB}I_{AB}\right]/P-\left(c+\delta_{B}\right)Z_{B}.\; \end{array}\right.$$

The plant model consists of four stages, S= density of healthy plants, and three different classes for infection, $I_{A}$, $I_{B}$ and $I_{AB}$, equal to the density of infected plants carrying only virus *A*, virus *B* or both viruses, respectively. The total plant population density is $P=S+I_{A}+I_{B}+I_{AB}$. The parameters for the plant model include σ= net planting rate, μ= per capita rate of harvesting/mortality, $\theta_{i}=$ probability an infective vector $Z_{i}$ inoculates a healthy plant with virus i=A,B per plant visit, ω= per capita recovery rate in a plant infected with a single virus, and $\omega_{i}=$ per capita viral recovery rate of virus *i* in a co-infected plant $I_{AB}$, i=A,B. The parameters $\gamma_{A}$ and $\gamma_{B}$ denote the synergistic or antagonistic interactions between the viruses within the plant when infective vectors inoculate a plant carrying a different virus, i.e., account for the probability of a second infection being either increased or reduced relative to a healthy plant. The plant model is

(2) $$\mathrm{Plant}\;\left\{\begin{array}{cc} \frac{dS}{dt} & =\sigma -\Phi S\left[\theta_{A}Z_{A}+\theta_{B}Z_{B}\right]/P-\mu S+\omega \left(I_{A}+I_{B}\right)\;\\ \frac{dI_{A}}{dt} & =\Phi \theta_{A}Z_{A}S/P-\Phi \gamma_{B}\theta_{B}Z_{B}I_{A}/P-\left(\mu +\omega \right)I_{A}+\omega_{A}I_{AB}\;\\ \frac{dI_{B}}{dt} & =\Phi \theta_{B}Z_{B}S/P-\Phi \gamma_{A}\theta_{A}Z_{A}I_{B}/P-\left(\mu +\omega \right)I_{B}+\omega_{B}I_{AB}\;\\ \frac{dI_{AB}}{dt} & =\Phi \left[\gamma_{B}\theta_{B}Z_{B}I_{A}+\gamma_{A}\theta_{A}Z_{A}I_{B}\right]/P-\left(\mu +\omega_{A}+\omega_{B}\right)I_{AB}.\; \end{array}\right.$$

The parameters $\epsilon_{i}$ and $\gamma_{i}$, i=A,B, reflect the fact that vector acquisition or inoculation with co-infection may differ from a single virus infection [24,25,30]. The acquisition of a single virus from a co-infected plant $I_{AB}$ may be greater or less than acquisition from a plant infected with a single virus (e.g., $\epsilon_{A}\alpha_{AB}>\alpha_{A}$ or $\epsilon_{A}\alpha_{AB}<\alpha_{A}$). In addition, the inoculation of a second virus into an infected plant may be greater or less than inoculation of a healthy plant (e.g., $\gamma_{B}\theta_{B}>\theta_{B}$ or $\gamma_{B}\theta_{B}<\theta_{B}$).

### 2.2. Invasion Thresholds

Two important disease threshold parameters are derived from models (1) and (2), the basic reproduction number and the invasion reproduction number. The density of non-infective vectors at the disease-free equilibrium (DFE) in the vector model (1) is $X=\Lambda /c=\bar{V}$ and the density of healthy plants at the DFE plant model (2) is $S=\sigma /\mu =\bar{P}$. The basic reproduction number can be computed from the next generation matrix approach [32,33,34,35,36] (Appendix A). Here it is defined as the maximum of two reproduction numbers,

(3) $$R_{0}=\max\left\{\frac{\Phi \bar{V}\alpha_{A}\Phi \theta_{A}}{\bar{P}\left(c+\delta_{A}\right)\left(\mu +\omega \right)},\frac{\Phi \bar{V}\alpha_{B}\Phi \theta_{B}}{\bar{P}\left(c+\delta_{B}\right)\left(\mu +\omega \right)}\right\}=\max\left\{R_{0A},R_{0B}\right\}.$$

The two terms in the preceding definition are basic reproduction numbers corresponding to infection with either virus *A* or virus *B*, $R_{0A}$ and $R_{0B}$, respectively. If the basic reproduction number for virus *A* exceeds the value of one, $R_{0A}>1,$ then virus *A* can invade the disease-free vector-plant system and if $R_{0B}>1$, then virus *B* can invade. An epidemiological interpretation of $R_{0A}$ is that if one vector inoculative with virus *A* is introduced into a healthy vector-plant system, it will inoculate and infect plants at a rate of $\Phi \theta_{A}$ during the period of time $1/(c+\delta_{A})$ the vector is infective. From an infected plant, a non-infective vector will acquire the virus at a rate $\Phi \alpha_{A}\bar{V}/\bar{P}$ during the period of time 1/(μ+ω) the plant is infected. If the product of these two expressions exceeds the value of one, then one infective vector (or infected plant) will generate more than one infective vector (or infected plant), resulting in an epidemic. In general, if $R_{0}>1$, then either virus *A* or *B* can invade the vector-plant system.

An invasion reproduction number can be derived if the system is already infected with a single virus. We consider whether virus *B* can invade when the system is at the virus *A* equilibrium, i.e., $R_{0A}>1$. The endemic equilibrium values for $\left(X,Z_{A},S,I_{A}\right)$ with virus *A* are $\left(\bar{V}-za_{eq},za_{eq},\bar{P}-ia_{eq},ia_{eq}\right)$. These endemic values can be expressed in terms of the basic reproduction number $R_{0A}$:

(4) $$za_{eq}=\frac{\left(R_{0A}-1\right)\bar{V}}{R_{0A}+\frac{\Phi \theta_{A}\bar{V}}{\bar{P}\left(\mu +\omega \right)}}\;\;\mathrm{and}\;\;ia_{eq}=\frac{\left(R_{0A}-1\right)\bar{P}}{R_{0A}+\frac{\Phi \alpha_{A}}{\left(c+\delta_{A}\right)}}.$$

To determine whether virus *B* can invade, we apply the next generation matrix approach to derive an invasion matrix $M_{invB}$ from which an invasion reproduction number $R_{invB}$ can be computed (Appendix B). We assume that the vector-plant system is at the virus *A* equilibrium, defined in (4), with the remaining states $Z_{B}$, $I_{B}$, and $I_{AB}$ set equal to zero. An invasion matrix for the system (1)–(2) is defined as follows:

(5) $$M_{invB}=\left(\begin{array}{ccc} 0 & \frac{\Phi \bar{X}\alpha_{B}}{\kappa_{1}} & \frac{\Phi \bar{X}\epsilon_{B}\alpha_{AB}}{\bar{P}\kappa_{2}}\;\\ \frac{\Phi \theta_{B}\bar{S}}{\bar{P}\left(c+\delta_{B}\right)} & 0 & \frac{\omega_{B}}{\kappa_{2}}\;\\ \frac{\Phi \gamma_{B}\theta_{B}ia_{eq}}{\bar{P}\left(c+\delta_{B}\right)} & \frac{\Phi \gamma_{A}\theta_{A}za_{eq}}{\kappa_{1}} & 0 \end{array}\right),$$

where $\bar{S}=\bar{P}-ia_{eq}$, $\bar{X}=\bar{V}-za_{eq}$, $\kappa_{1}=\left(\mu +\omega \right)\bar{P}+\Phi \gamma_{A}\theta_{A}za_{eq}$ and $\kappa_{2}=\mu +\omega_{A}+\omega_{B}$. A mathematical definition of the invasion reproduction number is the spectral radius of the invasion matrix, that is, $R_{invB}=\rho \left(M_{invB}\right)$. An epidemiological interpretation of $R_{invB}$ is the average number of new states, $Z_{B}$, $I_{B}$ or $I_{AB}$, that are produced after introduction of an average of one infective vector or infected plant containing virus *B*, $Z_{B}$, $I_{B}$ or $I_{AB}$, into the system infected with virus *A*. If $R_{invB}>1$, then virus *B* can invade and if $R_{invB}<1$, then virus *B* cannot invade. Each element in matrix $M_{invB}$ can be interpreted in terms of producing new infective vectors or infected plants, $Z_{B},I_{B}$ or $I_{AB}$. For example, the entry in the second row and first column,

$$\frac{\Phi \theta_{B}\bar{S}}{\bar{P}\left(c+\delta_{B}\right)},$$

can be interpreted as the average number of new infected plants $I_{B}$ that are produced when one infective vector $Z_{B}$ is introduced into the vector-plant system (where virus *A* has already invaded).

Since the invasion matrix (5) has non-negative entries, an increase (or a decrease) in any matrix entry also increases (or decreases) the invasion reproduction number [37]. In particular, if the inoculation probability $\theta_{B}$ for virus *B* increases so does the invasion reproduction number. The direction of change for the invasion reproduction number is not as straightforward if the vector visitation rate Φ or parameters related to virus *A* are changed, as the equilibrium values $ia_{eq}$ and $za_{eq}$ also change.

### 2.3. Formal Reduction to a Model That Does Not Track Vectors Explicitly

The vector-plant system can be reduced to a simplified plant model, where the vectors are not explicitly included (Appendix C). If the vector recovery rates, $\delta_{A}$ and $\delta_{B}$, are sufficiently large, then the vector population dynamics occur on a faster time scale than the plant population dynamics. The differential equations for the vector model (1) and the plant host model (2) can be approximated by another plant model without the vector variables:

(6) $$\begin{array}{c} \begin{array}{cc} \frac{dS}{dt} & =\sigma -\frac{\Phi^{2}\bar{V}}{P^{2}}S\left({\hat{\theta }}_{A}\left[\alpha_{A}I_{A}+\epsilon_{A}\alpha_{AB}I_{AB}\right]+{\hat{\theta }}_{B}\left[\alpha_{B}I_{B}+\epsilon_{B}\alpha_{AB}I_{AB}\right]\right)-\mu S+\omega \left(I_{A}+I_{B}\right)\\ \frac{dI_{A}}{dt} & =\frac{\Phi^{2}\bar{V}}{P^{2}}\left({\hat{\theta }}_{A}S\left[\alpha_{A}I_{A}+\epsilon_{A}\alpha_{AB}I_{AB}\right]-\gamma_{B}{\hat{\theta }}_{B}I_{A}\left[\alpha_{B}I_{B}+\epsilon_{B}\alpha_{AB}I_{AB}\right]\right)-\left(\mu +\omega \right)I_{A}+\omega_{A}I_{AB}\\ \frac{dI_{B}}{dt} & =\frac{\Phi^{2}\bar{V}}{P^{2}}\left({\hat{\theta }}_{B}S\left[\alpha_{B}I_{B}+\epsilon_{B}\alpha_{AB}I_{AB}\right]-\gamma_{A}{\hat{\theta }}_{A}I_{B}\left[\alpha_{A}I_{A}+\epsilon_{A}\alpha_{AB}I_{AB}\right]\right)-\left(\mu +\omega \right)I_{B}+\omega_{B}I_{AB}\\ \frac{dI_{AB}}{dt} & =\frac{\Phi^{2}\bar{V}}{P^{2}}\left(\gamma_{B}{\hat{\theta }}_{B}I_{A}\left[\alpha_{B}I_{B}+\epsilon_{B}\alpha_{AB}I_{AB}\right]+\gamma_{A}{\hat{\theta }}_{A}I_{B}\left[\alpha_{A}I_{A}+\epsilon_{A}\alpha_{AB}I_{AB}\right]\right)-\left(\mu +\omega_{A}+\omega_{B}\right)I_{AB}, \end{array} \end{array}$$

where parameters ${\hat{\theta }}_{i}=\theta_{i}/\left(c+\delta_{i}\right)$ for i=A,B. To distinguish the two sets of models, we will refer to the simplified plant model (6) as the vector-implicit model and to the vector-plant model (1) and (2) as the vector-explicit model.

The vector-implicit model retains the inoculation and acquisition terms. At the DFE, the basic reproduction number is the same as in (3). But the equilibrium for virus A $\left(\bar{S},{\bar{I}}_{A}\right)$ differs from the vector-explicit model,

(7) $$\bar{S}=\frac{\bar{P}}{R_{0A}}\;\;\mathrm{and}\;\;{\bar{I}}_{A}=\bar{P}\left(1-\frac{1}{R_{0A}}\right).$$

An invasion matrix for the vector-implicit model when $R_{0A}>1$ can be computed by a method similar to the computation of the invasion matrix in (5) (Appendix C). Invasion of virus *B* (via plants infected with virus *B*, $I_{B}$ or $I_{AB}$) is successful if the spectral radius of the following invasion matrix exceeds the value of one:

(8) $$M_{invB}=\left(\begin{array}{cc} \frac{\Phi^{2}\bar{V}\alpha_{B}{\hat{\theta }}_{B}\bar{S}}{\eta } & \frac{\Phi^{2}\bar{V}{\hat{\theta }}_{B}\alpha_{AB}\epsilon_{B}\bar{S}+{\bar{P}}^{2}\omega_{B}}{{\bar{P}}^{2}\left(\mu +\omega_{A}+\omega_{B}\right)}\\ \frac{\Phi^{2}\bar{V}{\bar{I}}_{A}\left[\gamma_{A}\alpha_{A}{\hat{\theta }}_{A}+\gamma_{B}\alpha_{B}{\hat{\theta }}_{B}\right]}{\eta }\; & \frac{\Phi^{2}\bar{V}\gamma_{B}{\hat{\theta }}_{B}\alpha_{AB}\epsilon_{B}{\bar{I}}_{A}}{{\bar{P}}^{2}\left(\mu +\omega_{A}+\omega_{B}\right)} \end{array}\right),$$

where $\eta ={\bar{P}}^{2}\left(\mu +\omega \right)+\Phi^{2}\bar{V}\gamma_{A}\alpha_{A}{\hat{\theta }}_{A}{\bar{I}}_{A}$.

## 3. Results

The effect of vector transmission on virus establishment in the vector-explicit model is examined in several numerical examples. In addition, the conditions for invasion of a second virus in the vector-explicit model are compared to the vector-implicit model.

### 3.1. Only One Virus Can Invade in Absence of the Other

**Figure 2:**
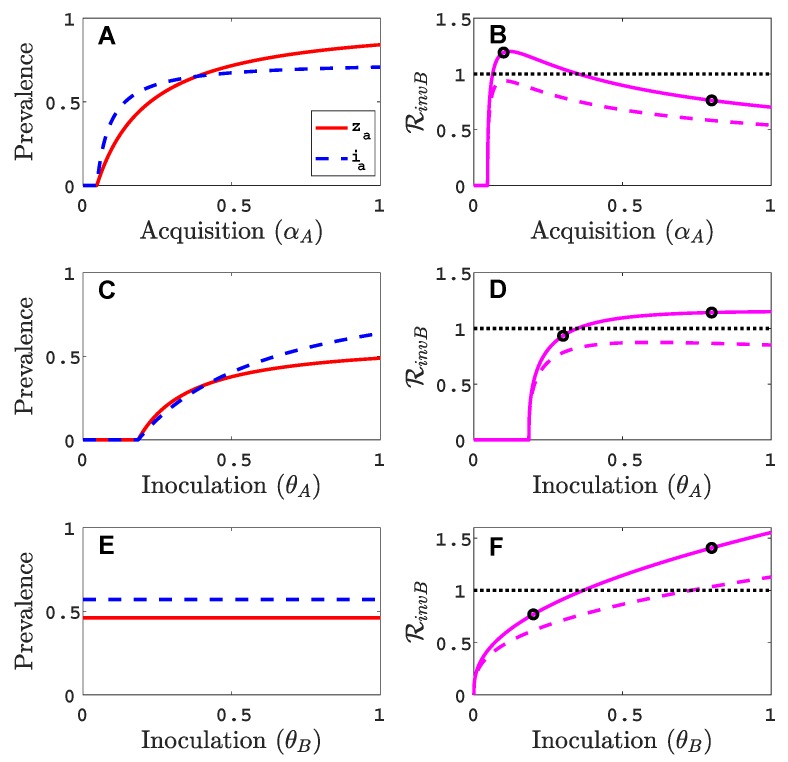
For the vector-explicit model, the virus *A* equilibrium prevalences for the vector $z_{a}=za_{eq}/\bar{V}$ and for the host $i_{a}=ia_{eq}/\bar{P}$ are graphed in panels (**A**,**C**,**E**) and the corresponding invasion reproduction numbers $R_{invB}$ are graphed in panels (**B**,**D**,**F**) as a function of acquisition ($\alpha_{A}$) or inoculation ($\theta_{A}$ and $\theta_{B}$) parameters. Virus *B* may invade the virus *A* equilibrium if the invasion reproduction number is greater than the threshold value one. The parameters values that are not varied are fixed at their default values (see Table 2): Λ=10 per month per unit area, Φ=30 per month, c=1 per month, $\alpha_{A}=0.2$, $\alpha_{B}=0$, $\alpha_{AB}=0.5$, $\epsilon_{A}=\epsilon_{B}=0.5$, $\delta_{A}=\delta_{B}=3$ per month, μ=1/12 per month, σ=1000μ (1000 per year per unit area), $\theta_{A}=0.8$, $\theta_{B}=0.5$ and $\omega =\omega_{A}=\omega_{B}=0$. Parameter values for the two invasion reproduction curves in (**B**,**E**,**F**) are $\gamma_{B}=0.9$ for the solid curve and $\gamma_{B}=0.25$ for the dashed curve. (**B**) The two black circles on the solid curve are at values of $\alpha_{A}=0.1,0.8$; (**D**) $\theta_{A}=0.3,0.8$; (**F**) $\theta_{B}=0.2,0.8$. The invasion reproduction number is $R_{invB}=\rho \left(M_{invB}\right)$ as defined in (5).

We assume that virus *A* can invade and persist in the vector-explicit model but not virus *B*. In particular, the acquisition probability of virus *B* is set to zero, $\alpha_{B}=0$, and all other acquisition and inoculation probabilities are positive. We investigate the invasion reproduction number as a function of the remaining acquisition and inoculation parameters, $\alpha_{A}$, $\theta_{A}$ and $\theta_{B}$, whose values lie in [0,1]. Table 2 is a summary of the default parameter values. The parameter values are chosen consistent with those summarised by Jeger et al. [5], but based on a time unit of one month. For each of the acquisition or inoculation parameters, Figure 2 shows a graph of the equilibrium prevalences for infective vectors and infected plants with virus *A* (with $z_{a}=za_{eq}/\bar{V}$ and $i_{a}=ia_{eq}/\bar{P}$) and the invasion reproduction curves. Two different invasion reproduction curves are graphed, one with $\gamma_{B}=0.9$ (solid invasion curve) and the other with $\gamma_{B}=0.25$ (dashed invasion curve). Parameter $\gamma_{B}$ denotes the inoculation success of virus *B* on a plant infected with virus *A*, relative to a healthy plant. Small values of $\gamma_{B}$ decrease the likelihood of a successful invasion of virus *B*.

As the acquisition probability $\alpha_{A}$ increases (Figure 2A,B), there is initially an increase in the invasion reproduction number but then it decreases as $\alpha_{A}$ approaches one. From the invasion matrix $M_{invB}$ in (5), it can be seen that some of the matrix entries increase and some decrease with an increase in $\alpha_{A}$, resulting in potential increases or decreases in the invasion reproduction number. The equilibrium prevalences $ia_{eq}$ and $za_{eq}$ increase with $\alpha_{A}$ which provide more opportunities for an infective vector $Z_{B}$ to inoculate infected plants $ia_{eq}$ and more opportunities for an infected plant $I_{B}$ to become inoculated from infective vectors $za_{eq}$. But the density of healthy plants, $\bar{S}$ in matrix (5), and the density of non-infective vectors, $\bar{X}$ in matrix (5), decrease with increases in $\alpha_{A}$ which provide fewer opportunities for $Z_{B}$ to inoculate healthy plants or for non-infective vectors to acquire virus *B* from a co-infected plant $I_{AB}$. For a relative inoculation success ratio of $\gamma_{B}=0.9$ (solid invasion curve), the invasion reproduction number exceeds the threshold value of one for a range of $\alpha_{A}$ values but for $\gamma_{B}=0.25$ (dashed invasion curve), the invasion reproduction number never exceeds the value of one.

**Figure A1:**
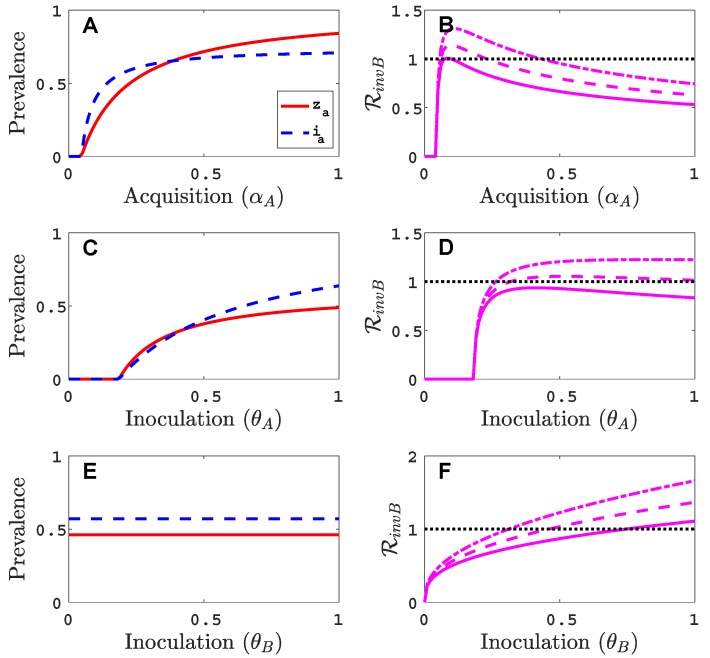
For the vector-explicit model, virus *A* equilibrium prevalences for the vector $z_{a}=za_{eq}/\bar{V}$ and for the host $i_{a}=ia_{eq}/\bar{P}$ are graphed in (**A**,**C**,**E**) and the corresponding invasion reproduction curves are graphed in (**B**,**D**,**F**) as a function of acquisition and inoculation parameters. The parameter values are the same as in Figure 2 with the exception that in panels B, D and F parameters $\gamma_{A}=2$ and $\gamma_{B}=0.1$ (solid curve), 0.5 (dashed curve), 1 (dashed-dot curve).

As the inoculation probability $\theta_{A}$ increases in Figure 2C,D, a similar effect on the invasion reproduction number could occur as for $\alpha_{A}$ but the decrease in the invasion reproduction number is not seen for these parameter values. The reduction in healthy plants and non-infective vectors is not as severe as for $\alpha_{A}$. Changes in the inoculation probability $\theta_{B}$ do not affect equilibrium prevalences $ia_{eq}$ and $za_{eq}$. Therefore, as $\theta_{B}$ increases, so does the invasion reproduction number (Figure 2E,F). Similarly, for large values of $\gamma_{B}$, the invasion reproduction number increases (Figure A1 in Appendix B).

**Figure A2:**
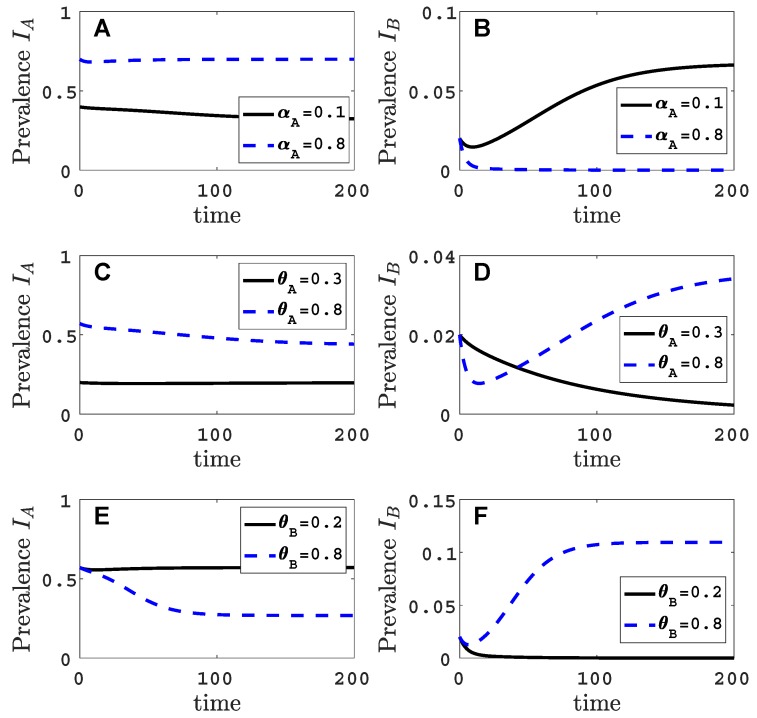
The time courses of one successful and one unsuccessful invasion of virus *B* are graphed for the vector-explicit model. The virus *A* host prevalences $I_{A}/P$ are graphed in panels (**A**,**C**,**E**) and the virus *B* host prevalences $I_{B}/P$ are graphed in panels (**B**,**D**,**F**) for the two sets of parameter values identified by black circles in Figure 2. The initial condition is perturbed slightly from the virus *A* equilibrium, $\left(\bar{V}-za_{eq}-0.1,va_{eq},0.1,\bar{P}-ia_{eq}-30,ia_{eq}-10,10,20\right)$. Parameter values for the graphs in panels (**A**–**F**) are the same as in Figure 2A–F with $\gamma_{B}=0.9$.

Parameter values in Figure 2, indicated by the black circles on the invasion curves, represent either a successful ($R_{invB}>1$) or an unsuccessful ($R_{invB}<1$) invasion of virus *B*. For these parameter values, the infection prevalence of viruses *A* and *B* in the plant population are graphed as functions of time in Figure A2 (Appendix B).

### 3.2. Comparison with Results of Model That Does Not Track Vectors Explicitly

**Figure 3:**
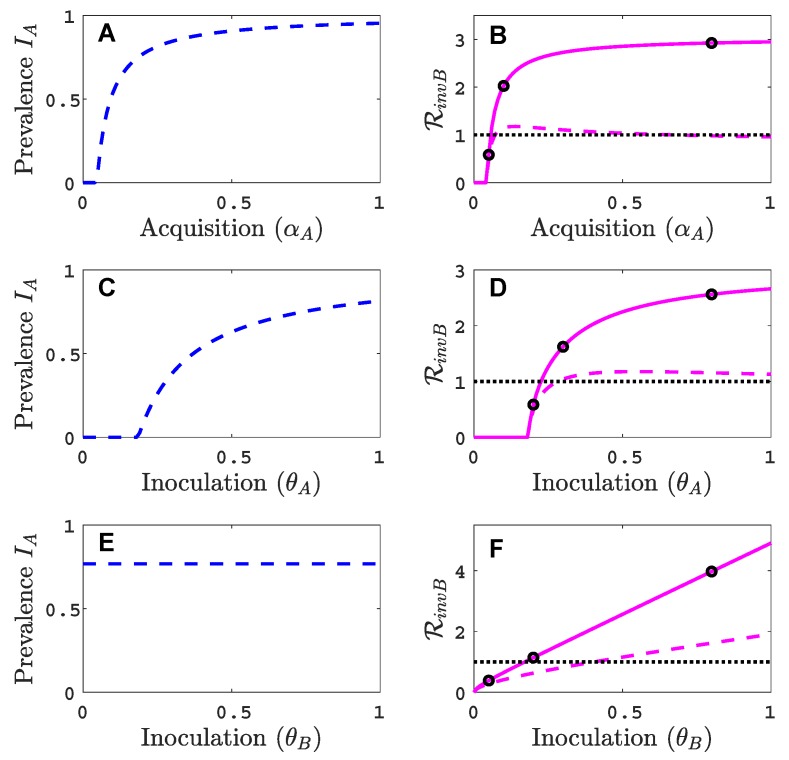
For the vector-implicit model, the virus *A* host equilibrium prevalences $ia_{eq}/\bar{P}$ are graphed in panels (**A**,**C**,**E**) and the corresponding invasion reproduction numbers $R_{invB}$ are graphed in panels (**B**,**D**,**F**) as a function of the acquisition ($\theta_{A}$) and inoculation ($\theta_{A}$ and $\theta_{B}$) parameters. The same parameter values are applied as in the vector-explicit model in Figure 2. The three black circles on the solid curve in (**B**) are at values of $\alpha_{A}=0.05,0.1,0.8$; in (**D**) $\theta_{A}=0.2,0.3,0.8$; (**F**) $\theta_{B}=0.05,0.2,0.8$. The invasion reproduction number equals $R_{invB}=\rho \left(M_{invB}\right)$ as defined in (8).

The dynamics of the vector-implicit model in Figure 3 are compared to the vector-explicit model when the same parameter values as in Figure 2 are applied. The differences in the invasion matrices and the virus *A* equilibrium values in the two models impact the invasion outcome. For the default parameter values in Table 2, the infected plant prevalences and the invasion reproduction values are larger in the vector-implicit model than in the vector-explicit model. The vector-implicit model predicts greater likelihood of co-infection than in the vector-explicit model. The differences may be attributed to the fact that the assumptions which led to the reduction to a plant model do not hold for all transmission classes. One of the assumptions in the vector-implicit model is that the infective vector life cycle is short, such as in non-persistent transmission. But in these examples, the parameter values for the vectors’ infective period, $1/\delta_{A}$ and $1/\delta_{B}$, are equal to 10 days, characteristic of persistent-circulative transmission [5]. These differences between the vector-explicit model and the vector-implicit model illustrate the importance of considering the mode of vector transmission in models of vectored plant viruses.

**Figure A3:**
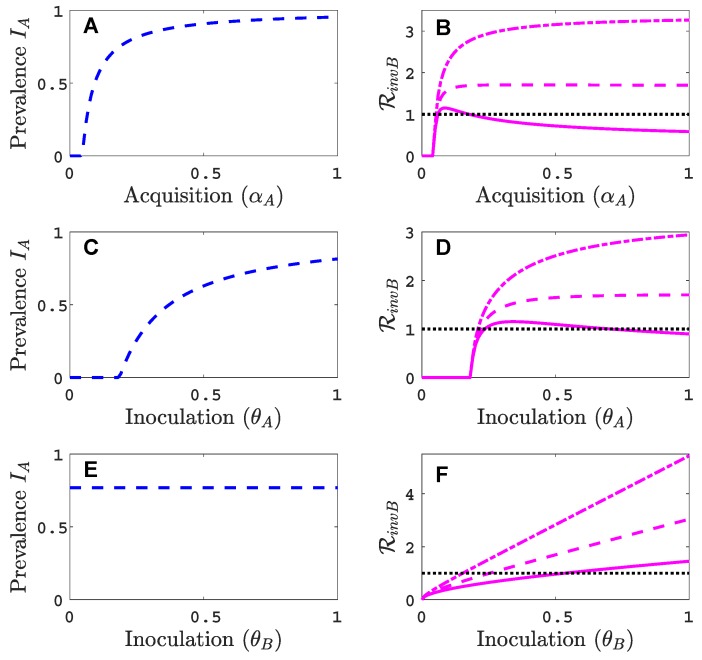
For the vector-implicit model, the virus *A* host equilibrium prevalences $i_{a}=ia_{eq}/\bar{P}$ are graphed in panels (**A**,**C**,**E**) and the corresponding invasion reproduction curves are graphed in panels (**B**,**D**,**F**) as a function of acquisition and inoculation parameters. Parameter values are as in Figure 3A–F with the exception that in panels B, D and F the parameter values for $\gamma_{A}=2$ and for $\gamma_{B}=0.1$ (solid curve), 0.5 (dashed curve), 1 (dashed-dot curve).

**Figure A4:**
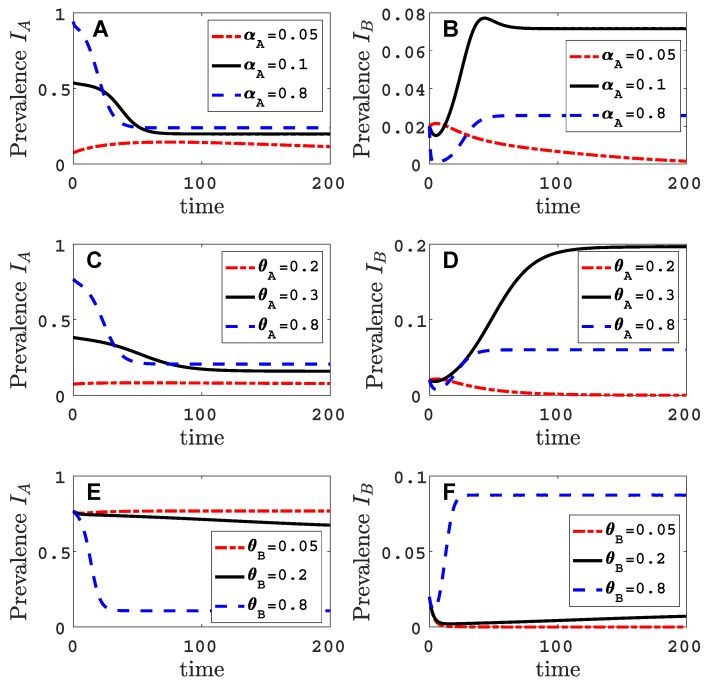
The time courses of two successful invasions and one unsuccessful invasion of virus *B* are graphed for the vector-implicit model. The virus *A* host prevalences $I_{A}/P$ are graphed in panels (**A**,**C**,**E**) and the virus *B* host prevalences $I_{B}/P$ are graphed in panels (**B**,**D**,**F**) for the three sets of parameter values identified by the black circles in Figure 3. The initial condition is perturbed slightly from the virus *A* equilibrium, $\left(\bar{P}-30,{\bar{I}}_{A}-10,10,20\right)$. Parameter values are as in Figure 3A–F with $\gamma_{B}=0.9$.

**Figure A5:**
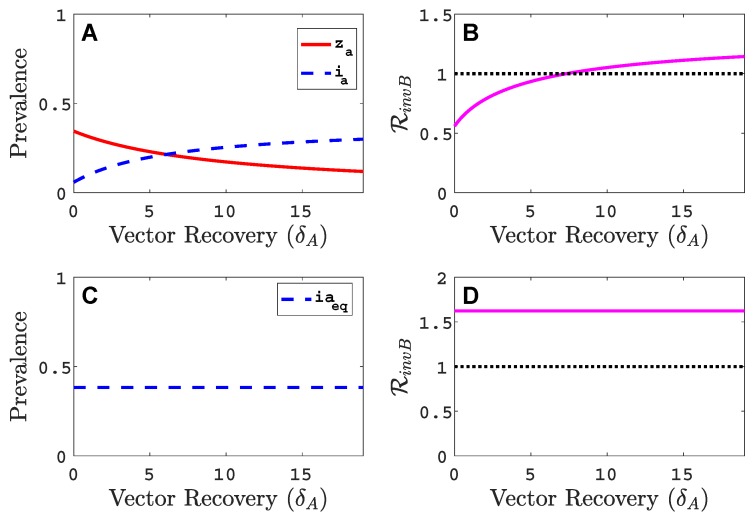
Comparison of the virus *A* equilibrium prevalences and invasion reproduction numbers in the vector-explicit model in (**A**,**B**) to the vector-implicit model in (**C**,**D**) as a function of vector recovery rate $\delta_{A}$. Parameter values are ${\hat{\theta }}_{A}=\theta_{A}/\left(c+\delta_{A}\right)=0.05$, $\alpha_{A}=0.3$, $\gamma_{B}=0.9$, $\delta_{A}\in \left[0,19\right]$, $\theta_{A}=0.05\left(c+\delta_{A}\right)\in \left[0.05,1\right]$ and other parameter values as in Table 2.

In Appendix C, additional invasion reproduction curves are graphed for different values of the relative inoculation success of viruses *A* and *B*, $\gamma_{A}$ and $\gamma_{B}$ in Figure A3 and as a function of vector recovery $\delta_{A}$ in Figure A5. Also, the infection prevalence of viruses *A* and *B* in the plant population for parameter values indicated by the black circles in Figure 3 are graphed as functions of time in Figure A4.

### 3.3. Neither Virus Can Invade in Absence of the Other

In this example, we assume neither virus can invade the vector-explicit model in the absence of the other virus, i.e., $R_{0A}<1$ and $R_{0B}<1$. There is only the disease-free equilibrium (DFE), which is always locally stable, and, for certain parameter values, we found numerically up to two co-infection equilibria. One of the latter is stable, so that the vector-explicit model is bistable. In the following analysis, we define $f_{a}$ and $f_{b}$ as the initial prevalences of virus *A* and virus *B* in the plant population, i.e., $f_{a}=\left(I_{A}+I_{AB}\right)/P\in \left[0,1\right]$ and $f_{B}=\left(I_{B}+I_{AB}\right)/P\in \left[0,1\right]$, respectively. For these prevalences, we will assume the following initial conditions of the plant population

(9) $$S\left(0\right)=\left(1-f_{a}\right)\left(1-f_{b}\right)\bar{P},\;I_{A}\left(0\right)=f_{a}\left(1-f_{b}\right)\bar{P},\;I_{B}\left(0\right)=f_{b}\left(1-f_{a}\right)\bar{P},\;\;\mathrm{and}\;\;I_{AB}\left(0\right)=f_{a}f_{b}\bar{P},$$

where $\bar{P}=\sigma /\mu$. For the initial conditions of the vector population, we assume

(10) $$X\left(0\right)=\left(1-\frac{f_{a}^{2}+f_{b}^{2}}{f_{a}+f_{b}}\right)\bar{V},\;Z_{A}\left(0\right)=\frac{f_{a}^{2}}{f_{a}+f_{b}}\bar{V},\;\;\mathrm{and}\;\;Z_{B}\left(0\right)=\frac{f_{b}^{2}}{f_{a}+f_{b}}\bar{V},$$

where $\bar{V}=\Lambda /c$, and which accounts for the fact that the initial prevalences of the viruses in the vectors cannot add beyond one because there is no co-infection.

**Figure 4:**
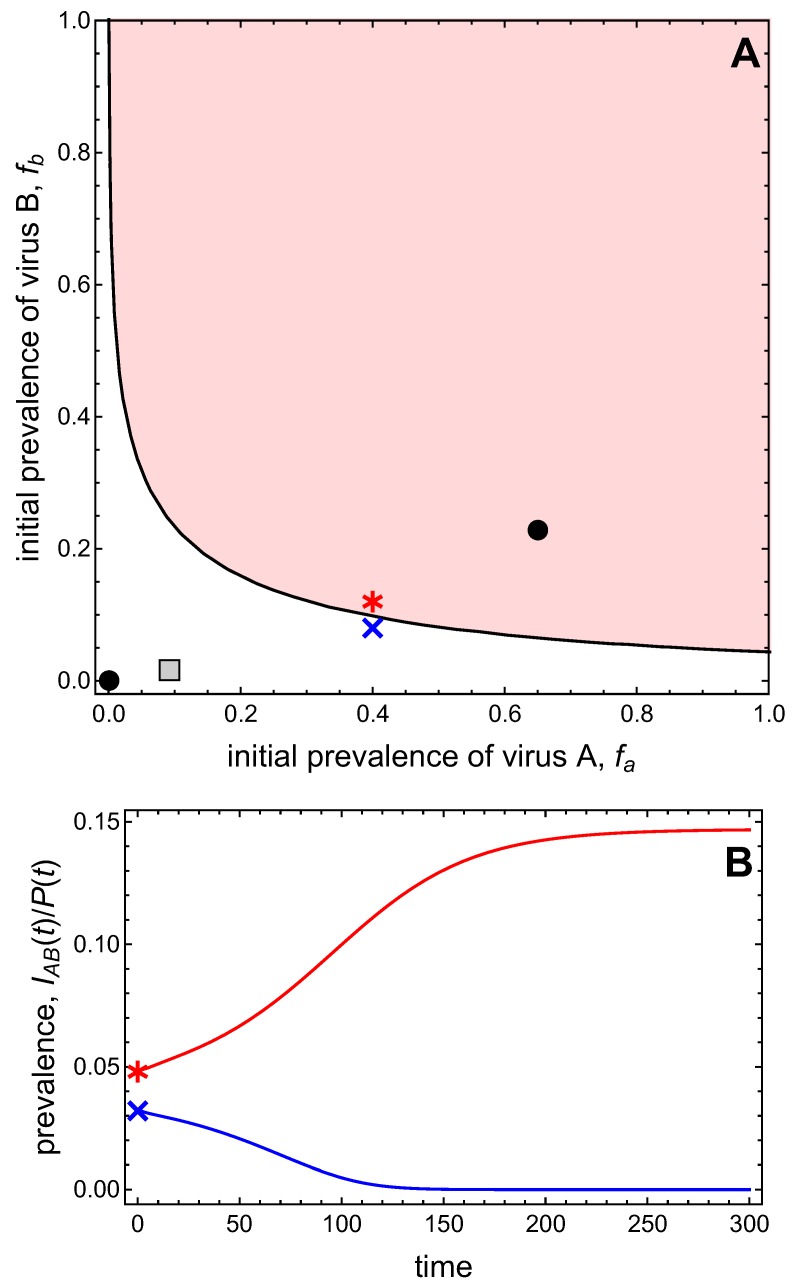
(**A**) The basins of attraction of the disease-free equilibrium (DFE) (white region) and the co-infected equilibrium (shaded region) are graphed as a function of the initial frequency $f_{a}$ of virus *A* and frequency $f_{b}$ of virus *B* in the plant population. See Equations (9) and (10) for the initial conditions. The points mark the prevalences of virus *A* and *B* in the plant population at the DFE (at the origin) and the stable co-infection equilibrium (in the interior). The gray square marks the relative frequency of virus *A* and *B* in the vector population at the stable co-infection equilibrium. The blue cross and red asterisk indicate initial conditions in different basins of attraction, for which time plots show convergence either to the DFE ($I_{AB}=0$) or to the co-infection equilibrium ($I_{AB}/P>0$) in (**B**). Parameter values are $\Lambda =1,\Phi =250,c=1,\alpha_{A}=0.005,\alpha_{B}=0.0005,\alpha_{AB}=0.15,\epsilon_{A}=\epsilon_{B}=0.5,\delta_{A}=20,\delta_{B}=40,\mu =1/12,\sigma =100\mu ,\theta_{A}=0.8,\theta_{B}=0.4,\omega =\mu /2=\omega_{A}=\omega_{B},\gamma_{A}=\gamma_{B}=0.5$ such that $R_{0A}=0.95$ and $R_{0B}=0.02$.

Figure 4A presents the bistable scenario and graphs the basins of attraction for the two stable equilibria as a function of the initial prevalences of virus *A* and virus *B*. In this graph, the origin represents the DFE, and the point representing the stable co-infection equilibrium indicates the virus prevalences at this equilibrium. The basin of attraction of an equilibrium is the set of initial conditions that will lead to that equilibrium in the long-run. As the system is bistable, there are two basins of attraction: one for the DFE which comprises smaller initial virus prevalences, and one for the co-infection state which comprises larger initial virus prevalences. The two basins of attraction are separated by a curve, the so-called separatrix. The time plots in Figure 4B illustrate that initial virus prevalences on one side of the separatrix approach the DFE in the long-run, while initial virus prevalences from the other side approach the co-infection state in the long-run.

**Figure 5:**
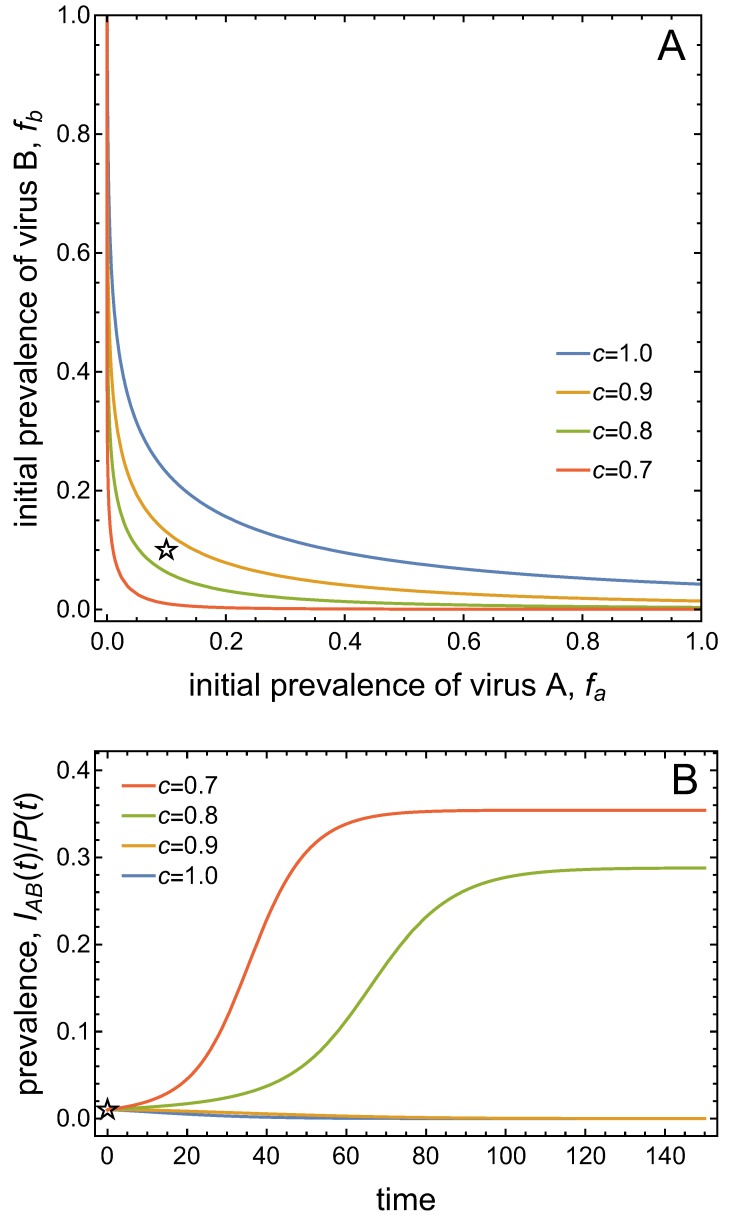
(**A**) The changes in the size of the basins of attraction for the DFE and the co-infected equilibrium are graphed as a function of the initial frequency $f_{a}$ of virus *A* and $f_{b}$ of virus *B* when the death rate of vectors decreases from c=1.0 to c=0.7, with values of c=0.7,0.8,0.9,1.0. The boundary separating the two basins of attraction at c=0.7 is closest to the origin = DFE and at c=1.0 is furthest from the origin. Other parameter values are as in Figure 4. The star symbol marks the initial condition for the disease progress curves in (**B**), which shows the time series of the prevalence of co-infected plants for varying levels of per-capita plant mortality, *c*. Initial conditions are fixed at (9) and (10) with initial virus prevalences $f_{a}=f_{b}=0.1$ in the plant population.

Figure 5A shows the separatrix for varying values of the vector mortality, *c*. The basin of attraction of the co-infection equilibrium expands in size when vector mortality is decreased. The size of the basin of attraction is also a measure of the resilience of the corresponding equilibrium, because the larger the basin of attraction, the more resistant the equilibrium against perturbations. That is, decreasing vector mortality enhances the resilience of the co-infection equilibrium, whereas increasing vector mortality enhances the resilience of the DFE. Now consider a given initial condition marked by the star in Figure 5A. Depending on the value of vector mortality, the same initial condition leads either to the disappearance of both viruses from the system (for larger vector mortalities) or to co-infection (for smaller vector mortalities). This is shown in Figure 5B. Notably, there is no gradual transition between these different outcomes. Instead, there is a rather drastic establishment of co-infection at high prevalence levels once both viruses can persist.

**Figure 6:**
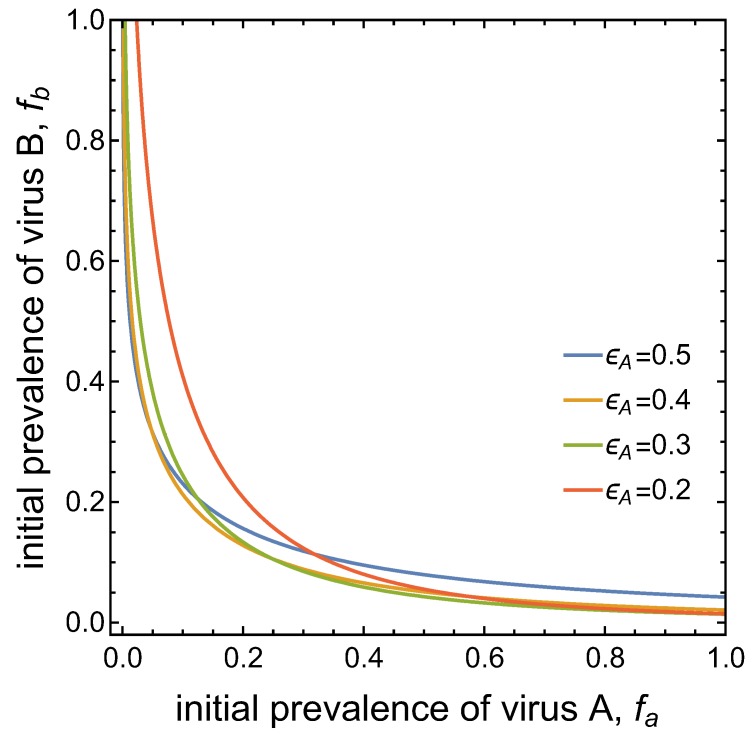
Basins of attraction for the DFE and the co-infection equilibrium for different values of $\epsilon_{A}$ and $\epsilon_{B}=1-\epsilon_{A}$. The blue curve with ϵ=0.5 corresponds to the separatrix shown in Figure 4 with $R_{0A}=0.95,R_{0B}=0.02$. Decreasing $\epsilon_{A}$, i.e. increasing $\epsilon_{B}$, reverses the asymmetry in the basins of attraction.

Note that the separatrix between the two basins of attraction is asymmetric for the set of parameter values considered thus far. This is due to the different transmission potentials of the two viruses, as expressed by the different values of $R_{0A}=0.95$ and $R_{0A}=0.02$ (when c=1). Figure 6 demonstrates that the ‘asymmetry’ in the basins of attraction can be ‘reversed’ when, for instance, the value of $\epsilon_{A}=1-\epsilon_{B}$ is decreased. This increases the conditional probability of vectors to acquire virus *B* from co-infected plants. That is, an advantage for virus *B* in the competition for vectors can compensate for the disadvantages of virus *B* assumed in the other parameter values.

## 4. Discussion

Co-infections are pervasive in plant virus epidemiology; yet, mathematical models keeping track of co-infections often leave vector dynamics implicit. This is understandable since (i) keeping track of co-infections makes the models less tractable mathematically, (ii) modeling vector dynamics explicitly may not be necessary relative to the research question addressed, and (iii) the biological knowledge or the data may be too scarce to reasonably increase the model’s complexity [38]. Moreover, it is well known [10,39,40] that vector-implicit models may reasonably approximate vector-explicit models when vector dynamics can be considered to be fast with respect to epidemiological dynamics in the plant host population (such as in non-persistent transmission). Although see [41] for a different model of non-persistent transmission of viruses that goes beyond differential equations to track the epidemiological effects of vector dynamics.

However, we have showed that vector-implicit and vector-explicit models may yield qualitatively different results (Figure 2 and Figure 3). The default parameter set we considered were appropriate for persistent-circulative transmission with vector infective periods of about 10 days (Table 2), and the qualitative difference in the models’ outcomes highlights a key biological feature of the vector-explicit model that is not accounted for in its vector-implicit analogue. This key feature is competition for vectors [16]. Competition occurs because we assumed one vector cannot be inoculative with two viruses at the same time at the point of inoculation. This assumption may reflect the fact that the first acquired virus saturates stylet receptor sites in non-persistent transmission, or that it precludes second inoculation of other viruses as long as the vector retains the first virus. The latter phenomenon would resemble the so-called “super-infection exclusion” usually associated with the host plant rather than with the vector [16]. Nevertheless, competitive exclusion (or pre-emptive competition) of one virus by another can also occur within vectors [42,43,44,45]. This way, infective vectors are only able to inoculate one virus at the point of inoculation. Therefore, viruses indirectly compete for vectors. However, the vector-implicit model is valid only if the vector recovery rate is sufficiently large, meaning that the vector loses the virus very quickly after acquisition (as is the case in non-persistent viruses). Therefore, it is very unlikely that a virus cannot be transmitted because the vector had already acquired another virus. These results highlight the importance of the mode of vector transmission in models of vectored plant viruses.

To illustrate the implications of our model, we considered two distinct special cases with two viruses (or virus strains) denoted *A* and *B*:

i Virus *A* is able to complete a full infection cycle, invade a disease-free population and settle at an endemic equilibrium in the absence of virus *B*. By contrast, virus *B* can systematically infect a plant but cannot be acquired by a vector in the absence of co-infection in the host plant. Successful invasion of virus *B* depends on a complex relation between vector acquisition and inoculation rates, the relative inoculation success of viruses *A* and *B* and the prevalence of infected hosts and infective vectors carrying virus *A*.
ii Virus *A* and virus *B* can both complete their infection cycles but are unable to invade a disease-free population in the absence of the other virus. The only biologically feasible endemic equilibrium is a co-infection equilibrium. The system will approach the co-infection equilibrium if the initial prevalences of virus *A* and *B* are sufficiently large, i.e., if the initial condition is in the basin of attraction of the co-infection equilibrium. Otherwise, if the initial virus prevalences are so low that the initial condition is in the basin of attraction of the disease-free equilibrium, both viruses will disappear from the system. The separatrix between these two different outcomes represents a curve of tipping points. On either side of these tipping points, we have contrasting dynamics, namely a disease-free versus a co-infected system. We have seen that increased vector mortality rates (e.g., due to vector control programs) moves the separatrix by making the co-infection equilibrium less resilient. This could lead to an abrupt (rather than gradual) extinction of co-infection.

In this paper, we have formulated a general epidemiological model for potential co-infection in a host plant by two virus species or strains. We used the model to investigate the effects of vector transmission on co-infection, specifically acquisition and inoculation, as well as antagonistic and synergistic interactions between viruses. We showed that reducing the model to a vector-implicit model can lose some of the key features gained when the vector is included explicitly (such as competition for vector). We derived a new invasion threshold that determines whether or not a second virus can invade a host population in which a first virus had successfully established. The invasion threshold highlights the key epidemiological parameters that are important for successful co-infection. However, the invasion threshold only applies near the virus *A* equilibrium when the invading virus *B* species/strain is at low prevalence levels. The dynamics of the models away from the virus *A* equilibrium can be quite complex and exhibit bistability, where both the virus *A* equilibrium and the co-infection equilibrium are stable. In this case, the initial prevalences of viruses *A* and *B* determine whether virus *B* can successfully invade. We also investigated the potential for co-infection and the conditions that need to be satisfied when one virus depends on an autonomous virus for its successful transmission or when both viruses are unable to invade alone. For persistent-circulative transmission, competition between viruses/virus strains, either direct or indirect, as they move through the vector is identified as a key and challenging area for further research that would improve modeling attempts to predict the epidemiological consequences of co-infection.

## Figures and Tables

**Table 1:** Summary of some representative experimental work on co-infection at different levels of complexity.

	Pathosystem	Authors	Key Message
**Two virus strains**	Potato virus Y (PYV)—*Myzus persicae* (host *Capsicum annuum*)	Moury et al. [15]	Two strains were equally transmissible and competition was studied to estimate the size of bottlenecks imposed by vector transmission. If there was a cost of virulence, modelling showed that virulent strains would go extinct.
	${\mathrm{PVY}}^{\mathrm{NTN}}$ compared with other strains—*Myzus persicae*	Srinivasan et al. [21]	Previous work had suggested some specificity in transmission of strains. The rate of infection for ${\mathrm{PVY}}^{\mathrm{NTN}}$ was higher than for other strains, a vector-related outcome as this was not observed with mechanical transmission.
	${\mathrm{PVY}}^{\mathrm{NTN}}$ compared with ${\mathrm{PVY}}^{O}$—*Myzus persicae*	Carroll et al. [22]	The necrotic strain was transmitted more efficiently than the wild-type. Co-infection would more likely result from inoculation by multiple aphids feeding on plants infected with the different strains rather than by single aphids feeding on multiple plants infected with the different strains.
**Two virus species—common vector**	Barley yellow dwarf virus/cereal yellow dwarf virus—*Rhopalosiphum padi*	Lacroix et al. [24]	The co-inoculation of BYDV-PAV lowered the CYDV-RPV infection rate but only at low nutrient supply rates. Broader environmental and nutritional factors can alter co-infection interactions and outcomes.
	Watermelon mosaic virus/zucchini yellow mosaic virus—*Aphis gossypii*	Salvaudon et al. [25]	ZYMV accumulated at similar rates in single and mixed infections, whereas WMV was much reduced in the presence of ZYMV. ZYMV also induced host changes that gave strong vector preference for infected plants; whereas WMV did not, although it was still readily acquired from mixed infections.
	Rice tungro spherical virus/rice tungro bacilliform virus—*Nephottetix virescens*	Holt and Chancellor [26]	Infection by each virus alone results in less pronounced symptoms. RTBV is retained in the vector for a longer period. When a vector carries both viruses, co-inoculation is common. When inoculative with RSTV alone the infection probability is higher.
**Two virus species—multiple vector species**	Bean pod mottle virus—*Epilachna varivestis*/soybean mosaic virus—*Aphis glycines*	Penaflor et al. [27]	Singly-infected plants with either BPMV or SMV increased soybean palatability, potentially enhancing acquisition of BPMV from BPMV plants and secondary infection of BPMV from SMV plants. BPMV infection had little effect on *A. glycines*, whereas SMV infection reduced aphid population growth but increased the preference for infected plants. With co-infection, effects on population growth were reversed and aphids showed a preference for co-infected plants.
**Multiple virus species—multiple vector species**	Grapevine leafroll-associated viruses (GLRaVs)—mealybugs/scale insects	Naidu et al. [28]	The exact role of GLRaVs in disease etiology remains unclear. With mealybugs, transmission is of a semi-persistent manner with a lack of vector-virus specificity.
		Blaisdell et al. [29]	Co-infections of GLRaVs are frequent in grapevines although with some spatial separation with implications for transmission and epidemiology.
	Sweet potato chlorotic stunt virus/sweet potato feathery mottle virus/multiple viruses—multiple vector species	Untiveros et al. [30]	Six viruses from the same or different virus families interacted synergistically with sweet potato virus disease, with increased disease symptoms, virus accumulation and movement in plants, and reduced yield of storage roots. All inoculations were made by grafting; no conclusions can be drawn on vector transmission effects.
**Multiple virus species—multiple vector species, multiple hosts**	Ecological networks formed by multiple co-infecting viruses in multiple hosts	McLeish et al. [31]	Co-infection networks were found to lead to strong non-random associations compared with single infections. Single infections were mostly related to habitat parameters, whereas co-infections were more related to ecological heterogeneity and ecosystem-level processes.

**Table 2:** Summary of model parameters for plants and vectors. All parameters are non-negative.

**Vector**		**Default Values**	**Default Values**
**Parameters**		**Section 3.1 and Section 3.2, Appendix B and Appendix C**	**Section 3.3**
Λ	vector birth rate	10/month/area	1/month/area
*c*	per capita vector natural death rate	1/month	1/month
Φ	number of plants visited/time by a vector	1/day	8.33/day
$\delta_{A}$	per capita infective vector recovery rate from virus *A*	3/month	0.66/day
$\delta_{B}$	per capita infective vector recovery rate from virus *B*	3/month	1.33/day
$\alpha_{A}$	probability non-infective vector acquires virus *A* from $I_{A}$ per plant visit	0.2	0.005
$\alpha_{B}$	probability non-infective vector acquires virus *B* from $I_{B}$ per plant visit	0	0.005
$\alpha_{AB}$	probability non-infective vector acquires a single virus, *A* or *B*, from $I_{AB}$ per plant visit	0.5	0.15
$\epsilon_{A}$	conditional probability of acquiring virus *A* from a co-infected plant $I_{AB}$, given a successful acquisition	0.5	0.5
$\epsilon_{B}$	conditional probability of acquiring virus *B* from co-infected plant $I_{AB}$, given a successful acquisition ($\epsilon_{B}=1-\epsilon_{A}$)	0.5	0.5
**Plant**		**Default Values**	**Default Values**
**Parameters**		**Section 3.1 and Section 3.2, Appendix B and Appendix C**	**Section 3.3**
μ	per capita mortality and or harvest of plants	1/year	1/year
σ	seeding or planting rate	1000/year/area	100/year/area
$\theta_{A}$	probability an infective vector with virus *A* inoculates a healthy plant per visit	0.8	0.8
$\theta_{B}$	probability an infective vector with virus *B* inoculates a healthy plant per visit	0.5	0.5
$\gamma_{A}$	relative inoculation success of virus *A* (as compared to a heathy plant) in a plant $I_{B}$, infected with a single virus *B*	0.9	0.5
$\gamma_{B}$	relative inoculation success of virus *B* (as compared to a healthy plant) in a plant $I_{A}$, infected with a single virus *A*	0.25, 0.9	0.5
ω	per-capita viral *A* or *B* loss rate in a plant infected with single virus	0	0.001/day
$\omega_{A}$	per-capita viral *B* loss rate (*A* is retained) from a co-infected plant $I_{AB}$	0	0.001/day
$\omega_{B}$	per-capita viral *A* loss rate (*B* is retained) from a co-infected plant $I_{AB}$	0	0.001/day

## Appendix A. Basic Reproduction Number

Linearization of the system of equations for the infected states $\left(Z_{A},I_{A},Z_{B},I_{B},I_{AB}\right)$ in models (1) and (2) and evaluated at the DFE, $X=\Lambda /c=\bar{V}$, $S=\sigma /\mu =\bar{P}$, with the remaining states equal to zero, yields the following Jacobian matrix:

$$J=\left(\begin{array}{ccccc} -c-\delta_{A} & \frac{\Phi \bar{V}\alpha_{A}}{\bar{P}} & 0 & 0 & \frac{\Phi \bar{V}\epsilon_{A}\alpha_{AB}}{\bar{P}}\\ \Phi \theta_{A} & -\mu -\omega & 0 & 0 & \omega_{A}\\ 0 & 0 & -c-\delta_{B} & \frac{\Phi \bar{V}\alpha_{B}}{\bar{P}} & \frac{\Phi \bar{V}\epsilon_{B}\alpha_{AB}}{\bar{P}}\\ 0 & 0 & \Phi \theta_{B} & -\mu -\omega & \omega_{B}\\ 0 & 0 & 0 & 0 & -\mu -\omega_{A}-\omega_{B} \end{array}\right).$$

The two 2×2 matrices on the diagonal are used to calculate $R_{0A}$ and $R_{0B}$. For example, the next generation matrix for virus *A* is

$$F_{A}V_{A}^{-1}=\left(\begin{array}{cc} 0 & \frac{\Phi \bar{V}\alpha_{A}}{\bar{P}\left(\mu +\omega \right)}\\ \frac{\Phi \theta_{A}}{c+\delta_{A}} & 0 \end{array}\right),$$

where

$$F_{A}=\left(\begin{array}{cc} 0 & \frac{\Phi \bar{V}\alpha_{A}}{\bar{P}}\\ \Phi \theta_{A} & 0 \end{array}\right)\;\;\mathrm{and}\;\;V_{A}=\left(\begin{array}{cc} c+\delta_{A} & 0\\ 0 & \mu +\omega \end{array}\right).$$

Matrix $F_{A}$ includes the rate of new infections and $V_{A}$ includes all of the other transfer rates. We define the basic reproduction number as

(A1) $$R_{0A}=\frac{\Phi \bar{V}\alpha_{A}\Phi \theta_{A}}{\bar{P}\left(c+\delta_{A}\right)\left(\mu +\omega \right)},$$

which is the product of the off-diagonal elements in the next generation matrix. The basic reproduction number is often defined as the spectral radius of the next generation matrix, the square root of this value, $\sqrt{R_{0A}}$ [36]. Both definitions have been applied in vector-host epidemic models [32,34,35,36]. Roberts and Heesterbeek [34] call the expression in (A1) the type reproduction number, whereas Heffernan et al. [32] call it the basic reproduction number and van den Driessche [35] calls it the target reproduction number. The terms “type” or “target” reproduction numbers are used in relation to a particular stage or type, where control measures may be targeted. The two expressions, with or without the square root, are equal at the threshold value of one. We choose the definition in (A1) for its simplicity and its straightforward epidemiological interpretation. The two expressions 1/(μ+ω) and $1/(c+\delta_{A})$ represent the average infective period of a plant or a vector, respectively. The entry in the first row and second column is the average number of infective vectors $Z_{A}$ produced by one infected plant $I_{A}$ during the plants’ infective period and the entry in the second row first column is the average number of infected plants $I_{A}$ produced by one infective vector $Z_{A}$ during the vectors’ infective period. Therefore,

$$R_{0A}=\frac{\Phi \bar{V}\alpha_{A}}{\bar{P}\left(\mu +\omega \right)}\times \frac{\Phi \theta_{A}}{c+\delta_{A}}.$$

## Appendix B. Invasion Reproduction Number

The system of equations for the infected states $\left(Z_{A},I_{A},Z_{B},I_{B},I_{AB}\right)$ are linearized about the virus A endemic equilibrium. This leads to the Jacobian matrix,

(A2) $$J=\left(\begin{array}{cc} J_{stab} & C\\ O & J_{invB} \end{array}\right).$$

Matrix $J_{stab}$ is a 2×2 stable matrix for the states $\left(Z_{A},I_{A}\right)$ (provided $R_{0A}>1$), *C* is a 2×3 matrix, *O* is a 3×2 zero matrix, and $J_{invB}$ is the invasion matrix for virus *B*. For simplicity, let $\bar{X}=\bar{V}-za_{eq}$ and $\bar{S}=\bar{P}-ia_{eq}$. The two diagonal block matrices in *J* are defined as

$$J_{stab}=\left(\begin{array}{cc} -\left(c+\delta_{A}\right)-\frac{\Phi \alpha_{A}ia_{eq}}{\bar{P}} & \frac{\Phi \bar{X}\alpha_{A}}{\bar{P}}\;\\ \frac{\Phi \theta_{A}\bar{X}}{\bar{P}} & -\left(\mu +\omega \right)-\frac{\Phi \theta_{A}za_{eq}}{\bar{P}} \end{array}\right)$$

and

$$J_{invB}=\left(\begin{array}{ccc} -c-\delta_{B} & \frac{\Phi \bar{X}\alpha_{B}}{\bar{P}} & \frac{\Phi \bar{X}\epsilon_{B}\alpha_{AB}}{\bar{P}}\;\\ \frac{\Phi \theta_{B}\bar{S}}{\bar{P}} & -\left(\mu +\omega \right)-\frac{\Phi \gamma_{A}\theta_{A}za_{eq}}{\bar{P}} & \omega_{B}\;\\ \frac{\Phi \gamma_{B}\theta_{B}ia_{eq}}{\bar{P}} & \frac{\Phi \gamma_{A}\theta_{A}za_{eq}}{\bar{P}} & -(\mu +\omega_{A}+\omega_{B}) \end{array}\right).$$

Invasion of virus *B* can be determined by computing the eigenvalues of matrix $J_{invB}$. Invasion is successful if matrix $J_{invB}$ is unstable, i.e., if matrix $J_{invB}$ has an eigenvalue with positive real part. However, computation of the three eigenvalues yield complicated expressions in terms of all of the parameters and do not give a simple criterion for invasion. An alternative is to compute an invasion reproduction matrix.

To compute the invasion reproduction number, we define $J_{invB}=F_{invB}-V_{invB}$. An invasion reproduction number is defined as the spectral radius of the matrix $F_{invB}V_{invB}^{-1}$,

(A3) $$R_{invB}=\rho \left(F_{invB}V_{invB}^{-1}\right).$$

If $R_{invB}>1$, then virus *B* can invade the system. Equivalent thresholds can also be defined using the next generation matrix approach, such as type or target reproduction numbers [34,35,36].

We define matrix $F_{invB}$ as the rate of new infections,

$$F_{invB}=\left(\begin{array}{ccc} 0 & \frac{\Phi \bar{X}\alpha_{B}}{\bar{P}} & \frac{\Phi \bar{X}\epsilon_{B}\alpha_{AB}}{\bar{P}}\;\\ \frac{\Phi \theta_{B}\bar{S}}{\bar{P}} & 0 & \omega_{B}\;\\ \frac{\Phi \gamma_{B}\theta_{B}ia_{eq}}{\bar{P}} & \frac{\Phi \gamma_{A}\theta_{A}za_{eq}}{\bar{P}} & 0 \end{array}\right),$$

and matrix $V_{invB}$ represents other transitions,

$$V_{invB}=\left(\begin{array}{ccc} c+\delta_{B} & 0 & 0\;\\ 0 & \mu +\omega +\frac{\Phi \gamma_{A}\theta_{A}za_{eq}}{\bar{P}} & 0\;\\ 0 & 0 & \mu +\omega_{A}+\omega_{B} \end{array}\right).$$

The recovery of virus *B* from a co-infected plant, $\omega_{B}$, is counted as a new infection in matrix $F_{invB}.$ Therefore, the invasion reproduction number is the spectral radius of the following matrix,

(A4) $$M_{invB}=F_{invB}V_{invB}^{-1}=\left(\begin{array}{ccc} 0 & \frac{\Phi \bar{X}\alpha_{B}}{\kappa_{1}} & \frac{\Phi \bar{X}\epsilon_{B}\alpha_{AB}}{\bar{P}\kappa_{2}}\;\\ \frac{\Phi \theta_{B}\bar{S}}{\bar{P}\left(c+\delta_{B}\right)} & 0 & \frac{\omega_{B}}{\kappa_{2}}\;\\ \frac{\Phi \gamma_{B}\theta_{B}ia_{eq}}{\bar{P}\left(c+\delta_{B}\right)} & \frac{\Phi \gamma_{A}\theta_{A}za_{eq}}{\kappa_{1}} & 0 \end{array}\right),$$

where $\kappa_{1}=\left(\mu +\omega \right)\bar{P}+\Phi \gamma_{A}\theta_{A}za_{eq}$ and $\kappa_{2}=\mu +\omega_{A}+\omega_{B}.$ The invasion reproduction number is the largest positive root of the characteristic equation of matrix $M_{invB},$ a cubic equation. Instead of writing the complex expression for the invasion reproduction number, we interpret how the nonzero entries in the invasion matrix $M_{invB}$ affect the invasion reproduction number.

Equilibrium prevalences of virus *A* in the plant population do not depend on the relative inoculation success of viruses *A* or *B*, parameters $\gamma_{A}$ or $\gamma_{B}$, but the invasion reproduction curves do depend on these parameters through the two matrix entries in (A4) in the last row. In Figure A1, three invasion reproduction curves are graphed for the relative inoculation success ratios $\gamma_{A}=2$ and for $\gamma_{B}=0.1,0.5,1.$ As $\gamma_{B}$ increases, so do the invasion curves.

Figure A2 illustrates the time course of a successful invasion ($R_{invB}>1$) and an unsuccessful invasion ($R_{invB}<1$) of virus *B*, corresponding to the two sets of parameter values indicated by the black circles on the solid invasion curve in Figure 2.

## Appendix C. Reduction to a Vector-Implicit Model

The vector dynamics generally occur on a faster time scale than the plant dynamics. We use this assumption to make a quasi-steady-state approximation for vector abundances [39]. Consider the vector model (1), where the total vector population is $V=X+Z_{A}+Z_{B}$. Assume that the vector recovery rates, $\delta_{A}$ and $\delta_{B}$, are large (short infective vector life cycle). Summing the differential equations for *X*, $Z_{A}$ and $Z_{B}$ leads to the following differential equation for *V*:

$$\frac{dV}{dt}=\Lambda -cV.$$

Thus, $V\left(t\right)=\Lambda /c+\left(V\left(0\right)-\Lambda /c\right)e^{-ct}$. For V(0)≈Λ/c, it follows that $V\left(t\right)\approx \Lambda /c=\bar{V}$ is approximately constant, an assumption applied in our model. Replace *X* by $\bar{V}-Z_{A}-Z_{B}$ in the differential equations for $Z_{A}$ and $Z_{B}$ and divide by $c+\delta_{A}$ or $c+\delta_{B}$:

$$\begin{array}{ccc} \epsilon_{1}\frac{dZ_{A}}{dt} & = & \frac{\Phi }{(c+\delta_{A})P}\left(\bar{V}-Z_{A}-Z_{B}\right)\left[\alpha_{A}I_{A}+\epsilon_{A}\alpha_{AB}I_{AB}\right]-Z_{A}\\ & \approx & \frac{\Phi }{(c+\delta_{A})P}\bar{V}\left[\alpha_{A}I_{A}+\epsilon_{A}\alpha_{AB}I_{AB}\right]-Z_{A}\approx 0\\ \epsilon_{2}\frac{dZ_{B}}{dt} & = & \frac{\Phi }{(c+\delta_{B})P}\left(\bar{V}-Z_{A}-Z_{B}\right)\left[\alpha_{B}I_{B}+\epsilon_{B}\alpha_{AB}I_{AB}\right]-Z_{B}\\ & \approx & \frac{\Phi }{(c+\delta_{B})P}\bar{V}\left[\alpha_{B}I_{B}+\epsilon_{B}\alpha_{AB}I_{AB}\right]-Z_{B}\approx 0. \end{array}$$

In particular, $\epsilon_{1}=1/\left(c+\delta_{A}\right)\ll 1$ and $\epsilon_{2}=1/\left(c+\delta_{B}\right)\ll 1$ due to the short life cycle of infective vectors. In addition, we assumed $\bar{V}\geq Z_{A}+Z_{B}$. This latter assumption holds if the vector visitation rate is much smaller than the vector death rate plus recovery rate $\Phi \ll (c+\delta_{i})$, i=A,B. Solving the right-hand sides of the differential equations for $Z_{A}$ and $Z_{B}$ leads to

(A5) $$\begin{array}{c} \begin{array}{cc} Z_{A} & \approx \frac{\Phi \bar{V}}{(c+\delta_{A})P}\left[\alpha_{A}I_{A}+\epsilon_{A}\alpha_{AB}I_{AB}\right]\\ Z_{B} & \approx \frac{\Phi \bar{V}}{(c+\delta_{B})P}\left[\alpha_{B}I_{B}+\epsilon_{B}\alpha_{AB}I_{AB}\right]. \end{array} \end{array}$$

Let ${\hat{\theta }}_{i}=\theta_{i}/\left(c+\delta_{i}\right)$ for i=A,B. The vector-implicit model (Equation (6) in the main text) is

$$\begin{array}{c} \begin{array}{cc} \frac{dS}{dt} & =\sigma -\frac{\Phi^{2}\bar{V}}{P^{2}}S\left({\hat{\theta }}_{A}\left[\alpha_{A}I_{A}+\epsilon_{A}\alpha_{AB}I_{AB}\right]+{\hat{\theta }}_{B}\left[\alpha_{B}I_{B}+\epsilon_{B}\alpha_{AB}I_{AB}\right]\right)-\mu S+\omega \left(I_{A}+I_{B}\right)\\ \frac{dI_{A}}{dt} & =\frac{\Phi^{2}\bar{V}}{P^{2}}\left({\hat{\theta }}_{A}S\left[\alpha_{A}I_{A}+\epsilon_{A}\alpha_{AB}I_{AB}\right]-\gamma_{B}{\hat{\theta }}_{B}I_{A}\left[\alpha_{B}I_{B}+\epsilon_{B}\alpha_{AB}I_{AB}\right]\right)-\left(\mu +\omega \right)I_{A}+\omega_{A}I_{AB}\\ \frac{dI_{B}}{dt} & =\frac{\Phi^{2}\bar{V}}{P^{2}}\left({\hat{\theta }}_{B}S\left[\alpha_{B}I_{B}+\epsilon_{B}\alpha_{AB}I_{AB}\right]-\gamma_{A}{\hat{\theta }}_{A}I_{B}\left[\alpha_{A}I_{A}+\epsilon_{A}\alpha_{AB}I_{AB}\right]\right)-\left(\mu +\omega \right)I_{B}+\omega_{B}I_{AB}\\ \frac{dI_{AB}}{dt} & =\frac{\Phi^{2}\bar{V}}{P^{2}}\left(\gamma_{B}{\hat{\theta }}_{B}I_{A}\left[\alpha_{B}I_{B}+\epsilon_{B}\alpha_{AB}I_{AB}\right]+\gamma_{A}{\hat{\theta }}_{A}I_{B}\left[\alpha_{A}I_{A}+\epsilon_{A}\alpha_{AB}I_{AB}\right]\right)-\left(\mu +\omega_{A}+\omega_{B}\right)I_{AB} \end{array} \end{array}$$

The vector parameters appear in the transmission coefficients. For example, the transmission coefficients from a co-infected plant $I_{AB}$ when $P=\bar{P}$ are

(A6) $$\beta_{i}=\frac{\Phi^{2}\bar{V}}{{\bar{P}}^{2}}{\hat{\theta }}_{i}\alpha_{AB},\;\;i=A,B.$$

The basic reproduction number and the invasion matrix for the plant model are calculated by a method similar to that used for the vector-explicit model. The basic reproduction number for the vector-implicit model is the same as in (3). The virus *A* equilibrium $\left(\bar{S},{\bar{I}}_{A}\right)$ is distinct from the equilibrium for the vector-explicit model in (4),

(A7) $$\bar{S}=\frac{\bar{P}}{R_{0A}}\;\;\mathrm{and}\;\;{\bar{I}}_{A}=\bar{P}\left(1-\frac{1}{R_{0A}}\right).$$

The invasion matrix for virus *B*, given $R_{0A}>1$, has the following form (matrix (8) in the main text):

$$M_{invB}=\left(\begin{array}{cc} \frac{\Phi^{2}\bar{V}\alpha_{B}{\hat{\theta }}_{B}\bar{S}}{\eta } & \frac{\Phi^{2}\bar{V}{\hat{\theta }}_{B}\alpha_{AB}\epsilon_{B}\bar{S}+{\bar{P}}^{2}\omega_{B}}{{\bar{P}}^{2}\left(\mu +\omega_{A}+\omega_{B}\right)}\\ \frac{\Phi^{2}\bar{V}{\bar{I}}_{A}\left[\gamma_{A}\alpha_{A}{\hat{\theta }}_{A}+\gamma_{B}\alpha_{B}{\hat{\theta }}_{B}\right]}{\eta }\; & \frac{\Phi^{2}\bar{V}\gamma_{B}{\hat{\theta }}_{B}\alpha_{AB}\epsilon_{B}{\bar{I}}_{A}}{{\bar{P}}^{2}\left(\mu +\omega_{A}+\omega_{B}\right)} \end{array}\right),$$

where $\eta ={\bar{P}}^{2}\left(\mu +\omega \right)+\Phi^{2}\bar{V}\gamma_{A}\alpha_{A}{\hat{\theta }}_{A}{\bar{I}}_{A}$. The invasion reproduction number $R_{invB}=\rho \left(M_{invB}\right)$ is the largest positive root of the quadratic characteristic equation of matrix $M_{invB}$. Alternately, a successful invasion can be assessed directly from the trace and determinant of the non-negative matrix $M_{invB}$ [46]. That is,

(A8) $$trace\left(M_{invB}\right)-det\left(M_{invB}\right)>1$$

if and only if $R_{invB}>1$.

Condition (A8) for invasion of virus *B* simplifies in the case that virus *B* cannot invade alone and there is no plant recovery, i.e., if $\alpha_{B}=0$ ($R_{0B}=0$) and in the plant population, $\omega =0=\omega_{A}=\omega_{B}$. Under these assumptions, condition (A8) can be expressed as

$$\frac{{\hat{\theta }}_{B}\epsilon_{B}\alpha_{AB}}{\alpha_{A}{\hat{\theta }}_{A}}\left(\gamma_{B}+\frac{\gamma_{A}}{1+\left(R_{0A}-1\right)\gamma_{A}}\right)\left(R_{0A}-1\right)>1.$$

The preceding condition shows that the invasion success of virus *B* increases if the parameters related to virus *B* are larger than those related to virus *A*, ${\hat{\theta }}_{B}\epsilon_{B}\alpha_{AB}>\alpha_{A}{\hat{\theta }}_{A}$, or if $\gamma_{B}$ is large.

For the parameter values $\alpha_{B}=0$ and $\omega =0=\omega_{A}=\omega_{B}$ and the same parameter values as in Figure A1, the equilibrium prevalences for plants infected with virus *A* and three invasion reproduction curves are graphed for the vector-implicit model in Figure A3. In Figure A4, the time courses of two successful invasions ($R_{invB}>1$) and one unsuccessful invasion ($R_{invB}<1$) are graphed for the vector-implicit model and for parameter values as in Figure 3.

If the vector recovery rate $\delta_{A}$ is increased (infective period of vector is shortened) there is better agreement between the vector-implicit and vector-explicit models. That is, if the vector-explicit model predicts no invasion of virus *B* so does the vector-implicit model and vice versa. In addition, the values of the host equilibrium prevalences for the two models are in closer agreement for large values of $\delta_{A}$. We can see this if we fix the composite parameter ${\hat{\theta }}_{A}=\theta_{A}/\left(c+\delta_{A}\right)=K=$ constant in the vector-implicit model but vary $\theta_{A}$ and $\delta_{A}$. The virus *A* prevalence and the invasion reproduction number in the vector-implicit model does not change as $\delta_{A}$ and $\theta_{A}$ change but this is not the case for the virus *A* prevalence and the invasion reproduction number in the vector-explicit model (see Figure A5). The invasion reproduction number in the vector-implicit model predicts species *B* invasion success $R_{invB}>1$ but in the vector-explicit model, a successful invasion occurs only if $\delta_{A}$ is sufficiently large. In the example in Figure A5, K=0.05, $\gamma_{B}=0.9,$ and $\alpha_{A}=0.3$ with other parameter values as in Table 2. Panels A and B in Figure A5 are the results for the vector-explicit model and panels C and D are for the vector-implicit model. The invasion curve where $R_{invB}>1$ in the vector-explicit model predicts a successful invasion only if $\delta_{A}>7.$
